# Predictive Modeling of Acute Respiratory Distress Syndrome Using Machine Learning: Systematic Review and Meta-Analysis

**DOI:** 10.2196/66615

**Published:** 2025-05-13

**Authors:** Jinxi Yang, Siyao Zeng, Shanpeng Cui, Junbo Zheng, Hongliang Wang

**Affiliations:** 1 The Second Clinical Medical College Harbin Medical University Heilongjiang Province, Harbin China; 2 Department of Critical Care Medicine The Second Affiliated Hospital of Harbin Medical University Heilongjiang Province, Harbin China

**Keywords:** acute respiratory distress syndrome, machine learning, predictive modeling, systematic evaluation, ICU, detection, prediction models, effectiveness, patient outcomes, PRISMA

## Abstract

**Background:**

Acute respiratory distress syndrome (ARDS) is a critical condition commonly encountered in the intensive care unit (ICU), characterized by a high incidence and substantial mortality rate. Early detection and accurate prediction of ARDS can significantly improve patient outcomes. While machine learning (ML) models are increasingly being used for ARDS prediction, there is a lack of consensus on the most effective model or methodology. This study is the first to systematically evaluate the performance of ARDS prediction models based on multiple quantitative data sources. We compare the effectiveness of ML models via a meta-analysis, revealing factors affecting performance and suggesting strategies to enhance generalization and prediction accuracy.

**Objective:**

This study aims to evaluate the performance of existing ARDS prediction models through a systematic review and meta-analysis, using metrics such as area under the receiver operating characteristic curve, sensitivity, specificity, and other relevant indicators. The findings will provide evidence-based insights to support the development of more accurate and effective ARDS prediction tools.

**Methods:**

We performed a search across 6 electronic databases for studies developing ML predictive models for ARDS, with a cutoff date of December 29, 2024. The risk of bias in these models was evaluated using the Prediction model Risk of Bias Assessment Tool. Meta-analyses and investigations into heterogeneity were carried out using Meta-DiSc software (version 1.4), developed by the Ramón y Cajal Hospital’s Clinical Biostatistics team in Madrid, Spain. Furthermore, sensitivity, subgroup, and meta-regression analyses were used to explore the sources of heterogeneity more comprehensively.

**Results:**

ML models achieved a pooled area under the receiver operating characteristic curve of 0.7407 for ARDS. The additional metrics were as follows: sensitivity was 0.67 (95% CI 0.66-0.67; *P*<.001; *I*²=97.1%), specificity was 0.68 (95% CI 0.67-0.68; *P*<.001; *I*²=98.5%), the diagnostic odds ratio was 6.26 (95% CI 4.93-7.94; *P*<.001; *I*²=95.3%), the positive likelihood ratio was 2.80 (95% CI 2.46-3.19; *P*<.001; *I*²=97.3%), and the negative likelihood ratio was 0.51 (95% CI 0.46-0.57; *P*<.001; *I*²=93.6%).

**Conclusions:**

This study evaluates prediction models constructed using various ML algorithms, with results showing that ML demonstrates high performance in ARDS prediction. However, many of the existing models still have limitations. During model development, it is essential to focus on model quality, including reducing bias risk, designing appropriate sample sizes, conducting external validation, and ensuring model interpretability. Additionally, challenges such as physician trust and the need for prospective validation must also be addressed. Future research should standardize model development, optimize model performance, and explore how to better integrate predictive models into clinical practice to improve ARDS diagnosis and risk stratification.

**Trial Registration:**

PROSPERO CRD42024529403; https://www.crd.york.ac.uk/PROSPERO/view/CRD42024529403

## Introduction

Acute respiratory distress syndrome (ARDS) is a heterogeneous condition characterized by diffuse lung inflammation and edema, affecting 10% of intensive care unit (ICU) admissions and 23% of mechanically ventilated patients, with mortality rates up to 45% [1]. The Berlin definition, widely used for ARDS diagnosis, faced challenges during the COVID-19 pandemic, particularly its positive end-expiratory pressure ≥5 cm H_2_O requirement, as many patients on noninvasive ventilation did not meet this criterion. In 2023, the European Society of Intensive Care Medicine updated its guidelines to broaden the ARDS definition and improve diagnostic tools [2]. Despite these advancements, early prediction and management of ARDS remain challenging due to its clinical complexity and heterogeneity.

In recent years, machine learning (ML) has emerged as a highly promising tool in the medical field, particularly in the areas of early diagnosis and enhancing clinical decision-making [3]. The field has witnessed rapid advancements, especially after 2020, with advanced models, such as transformer architectures, multimodal ML, explainable ML, and reinforcement learning techniques, achieving significant improvements in both performance and efficiency [4-7]. These advancements have not only provided a new technological foundation for our research but also prompted us to re-evaluate existing methodologies. ML focuses on how to use data to improve system performance through computational methods [8]. Previous studies have demonstrated that both supervised and unsupervised ML techniques can be applied to build risk models and refine patient categorization [9]. However, there are several critical gaps in the current literature. For example, some ARDS prediction studies, being small-sample and single-center, have reduced the generalizability of their models, limiting their applicability to broader patient populations [10]. Additionally, variations in data extraction and preprocessing methods, inconsistent evaluation metrics (eg, AUC and sensitivity), and suboptimal model optimization techniques hinder the comparability and performance of ML models.

A 2024 meta-analysis reviewed over a dozen ML algorithms for ARDS prediction but found no consensus on the best-performing approach across diverse clinical scenarios [11]. Furthermore, few studies have comprehensively evaluated multiple data sources to assess model performance, highlighting the need for systematic comparisons.

This study aims to address the existing gaps through a systematic review and meta-analysis of ARDS prediction models. We followed a standardized process, sequentially conducting data extraction and quality assessment, and compared the predictive performance of different ML algorithms using metrics such as AUC, sensitivity, and specificity. Through subgroup analysis and meta-regression, we identified sources of heterogeneity and explored key factors influencing model performance. Based on these findings, we provided specific implementation strategies for developing reliable and generalizable ARDS prediction models.

## Methods

### Research Design

This study was carried out in adherence to Preferred Reporting Items for Systematic Reviews and Meta-Analyses (PRISMA) guidelines (Multimedia Appendix 1) [12].

### Search Methods

A thorough literature search was performed across 6 databases (PubMed, Web of Science, ProQuest, Scopus, Embase, and Cochrane) up to December 29, 2024, to locate studies investigating the use of ML in predicting and diagnosing ARDS. Search terms included combinations of “artificial intelligence,” “deep learning,” “machine learning,” “neural networks, computer,” “acute respiratory distress syndrome,” and “prediction.” For a detailed search strategy, refer to Multimedia Appendix 2.

#### Inclusion and Exclusion Criteria

Studies were included based on the following criteria: (1) published in English and (2) involving a study population of adults aged 18 years and older; (3) the diagnosis of ARDS was confirmed; (4) the study developed or updated a predictive model; (5) the study presented at least one validated ML predictive model; (6) the study adequately evaluated the performance of the model, providing data to derive sensitivity and specificity; and (7) the study encompassed both prospective and retrospective cohort studies, along with control groups from pertinent randomized controlled trials.

Studies were excluded if (1) the study population included individuals younger than 18 years, (2) the article was not published in English, and (3) the article was a case report or review or the article failed to provide adequate data.

#### Data Extraction

Data extraction and screening were conducted by JXY and SYZ in accordance with the transparent reporting of a multivariable prediction model for individual prognosis or diagnosis standardized protocol [13]. Any disagreements were resolved through consensus with the involvement of a third researcher, SPC. The level of agreement between the 2 researchers was assessed using the Cohen κ statistic. In total, 17 studies were selected for analysis. The extracted data from each study included (1) demographic information; (2) methods for data segmentation, feature selection techniques, ML algorithms, and model validation and application; and (3) prediction outcomes such as accuracy, sensitivity, specificity, and area under the receiver operating characteristic curve (AUROC).

#### Assessment of Bias

The Predictive modeling Risk of Bias Assessment Tool (PROBAST) was used to assess the risk of bias (ROB) and the applicability of the included literature. PROBAST consists of 4 domains (study population, predictors, outcomes, and statistical analyses), comprising a total of 20 questions [14].

#### Statistical Analysis

The performance of a prediction model is evaluated through 2 key metrics: calibration and discrimination. Discriminative power is assessed by the AUROC value, while model calibration assesses the agreement between observed and predicted outcomes, often visualized through calibration plots [15]. The Meta-DiSc (version 1.4) software program was used in this meta-analysis to calculate pooled estimates of AUROC, sensitivity, specificity, positive likelihood ratio (PLR), negative likelihood ratio (NegLR), and diagnostic odds ratio (DOR) [16]. This software allows for the combination of effect sizes, examination of heterogeneity, and assessment of threshold effects. The *I*² statistic assessed statistical heterogeneity among the included studies, with an *I*² value above 75% indicating significant heterogeneity [17]. The study included both primary and subgroup analyses to evaluate the performance of different ML methods for predicting ARDS in clinical settings. Sensitivity and subgroup analyses were also performed to identify potential sources of heterogeneity. The subgroup analysis focused on comparing the predictive capabilities of different ML algorithms (eg, logistic regression, Bayesian modeling, and artificial neural networks [ANNs]) in ARDS prediction, while the sensitivity analysis assessed the robustness of the study’s findings. Regression analysis is used to explore and explain the heterogeneity between different study results. It can help reveal potential influencing factors and analyze which variables might affect the effect size of each study.

## Results

### Selection of Studies

**Figure 1:**
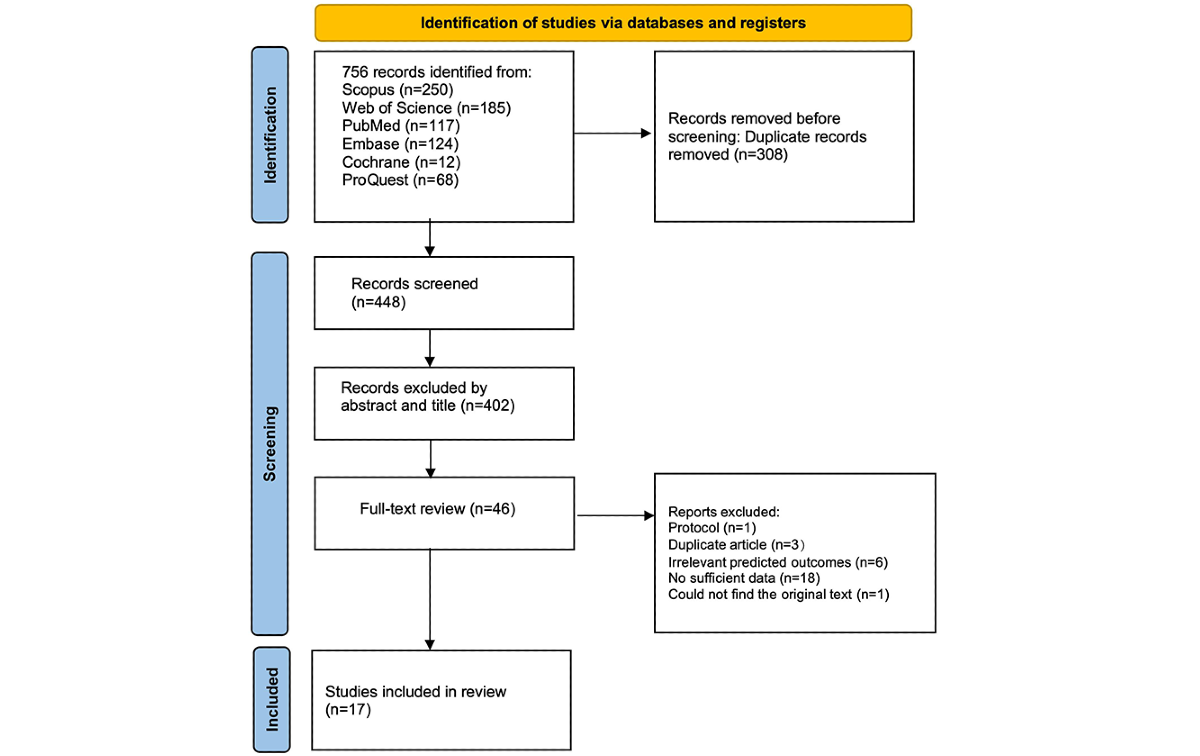
PRISMA flow diagram for study selection.

The search initially yielded 756 records. After duplicates were removed, 448 records remained. Titles and abstracts were then screened, resulting in the evaluation of 46 articles based on inclusion criteria. Ultimately, 17 studies were included in the meta-analysis [18-34]. Cohen κ was used to assess the agreement between the 2 researchers during the literature screening process. The resulting value was 0.818, indicating strong agreement between the researchers (>0.8 is considered strong agreement). The PRISMA flowchart illustrating the study screening process is shown in Figure 1.

### Characteristics of the Studies

A total of 17 studies [18-34], involving 148,384 patients, were included in the analysis; all were published within the last 10 years, with 2 published in 2024. In total, 9 studies [18,22-25,27,31,33,34] focused on ICU patients, 6 [19,21,28,30,32] on hospitalized patients, and 2 [26,29] included both ICU and hospitalized patients. In total, 4 studies [18,23,28,30] examined patients with COVID-19 infection, while another 4 [19-21,31] focused on patients with acute pancreatitis. In total, 64 models were systematically evaluated. Table 1 provides detailed information on the characteristics of the included studies.

**Table 1:** Study characteristics.

Author, year	Sample size	Model type	Data source	ARDS^a^ definition or identification	Department	External validation	Outcomes (top models and predictors)
Zhou et al [18], 2023	103	XGBoost^b^ CNN^c^	Shanghai Renji Hospital	Berlin definition	ICU^d^	No	XGBoost Age PaO_2_/FiO_2_^e^ CRP^f^ The count of total T-lymphocytes Interleukin-6
Zou et al [19], 2023	214	ANN^g^ LR^h^	Affiliated Hospital of Southwest Medical University	Berlin definition	Hospitalized patient	No	ANN BISAP^i^ score PCT^j^ PT^k^ Ca2+
Zhang and Pang [20], 2023	460	SVM^l^ EDTs^m^ BC^n^ LR	Xuanwu Hospital of Capital Medical University	Berlin definition	Hospitalized patient	No	BC PaO_2_ CRP NLR^o^ Ca2+ WBC^p^
Zhang et al [21], 2023	440	LR RF^q^ SVM DT^r^ XGBoost	Zhongda hospital	Berlin definition	Hospitalized patient	No	XGBoost PaO_2_/FiO_2_ APACHE II^s^ SOFA^t^ K^+^^u^ AMY^v^
Wang et al [22], 2023	649	XGBoost LightGBM^w^ RF AdaBoost^x^ CNB^y^ SVM	MIMIC-III^z^ database	Berlin definition	ICU	No	RF Age Systolic blood pressure Heart rate Abbreviated Injury Score chest, etc
Singhal et al [23], 2021	14,785	XGBoost	Cerner real-world data Emory Healthcare UTHSC^aa^-Methodist LeBonheur Healthcare	Berlin definition	ICU	Yes	XGBoost SpO_2_^ab^ (minimum) Systolic blood pressure Age FiO_2_ (max) Respiratory rate (max), etc
Mo et al [24], 2023	989	ANN LR	Changzhou Second People's Hospital	Berlin definition	ICU and the respiratory and critical care medicine departments	No	ANN LDH^ac^ APTT^ad^ PCT Age MRR^ae^, etc
Marshall et al [25], 2022	2078	XGBoost	Emory University Healthcare	Berlin definition	ICU	No	XGBoost Presence of the ventilator Total time on ventilation Tidal volume Waveform features
Lam et al [26], 2022	40,703	XGBoost RNN^af^	7 Hospital	Berlin definition	Emergency department inpatient ward and ICU	Yes	RNN Monocytes SpO_2_
Bai et al [27], 2022	31,184	NB^ag^ LR XGBoost AdaBoost RF	Telehealth Intensive Care Unit MIMIC-IV^ah^	ICD-9^ai^ ICD-10^aj^	ICU	Yes	AdaBoost APACHE IV minimum and maximum HCO_3_^ak^ lactate creatinine albumin, etc
Xu et al [28], 2021	659	LR RF SVM DT DNN^al^	Wuhan and non-Wuhan areas which were confirmed with COVID-19	Berlin definition	Hospitalized patient	Yes	DT Severity evaluation at admission Gender Age BMI Temperature, etc
Lam et al [29], 2021	29,127	RNN	7 US hospitals	*ICD-9*	Emergency department inpatient ward and ICU ICU	Yes	RNN Systolic blood pressure Respiratory rate
Izadi et al [30], 2022	8633	KNN^am^ SVM GLMNET^an^ BAYESGLM^ao^ GAM^ap^ GBM^aq^ NN^ar^	COVID‐19 Global Rheumatology Alliance Registry	Diagnosed by multiple experts	Hospitalized patient	Yes	GBM Age Higher average daily prednisone‐equivalent glucocorticoid doses Pulmonary hypertension Interstitial lung disease Chronic renal insufficiency or end‐stage renal disease Anti‐CD20 monoclonal antibody use, etc
Fei et al [31], 2018	217	ANN LR	Jinling Hospital	Berlin definition	Surgical Intensive Care Unit	No	ANN PNR^as^ LDH SaO_2_%^at^
Zhang et al [32], 2024	1996	DT GBDT^au^ XGBoost AdaBoost LightGBM RF Deep forest	NanjingFirst Hospital Shanghai General Hospital	Berlin definition	Hospitalized patient	Yes	DT Chronic obstructive pulmonary disease Preoperative albumin Central venous pressure Cardiopulmonary bypass time Left ventricular ejection fraction
Lin et al [33], 2024	11,409	KNN XGBoost SVM DNN^av^ DT	MIMIC-IV database	Oxygenation index<300	ICU	Yes	XGBoost Oxygenation index PaO_2_ Hematocrit Heart rate Mean arterial pressure, etc
Wu et al [34], 2022	4738	LightGBM AdaBoost LR RF Naïve Bayes KNN SVM	Telehealth Intensive Care Unit Collaborative Research Database	Berlin definition	ICU	No	LightGBM SpO_2_

^a^ARDS: acute respiratory distress syndrome.

^b^XGBoost: extreme gradient boosting.

^c^CNN: convolutional neural network.

^d^ICU: intensive care unit.

^e^PaO_2_/FiO_2_: arterial partial pressure of oxygen/fractional inspired:oxygen ratio.

^f^CRP: C-reactive protein.

^g^ANN: artificial neural network.

^h^LR: logistic regression.

^i^BISAP: Bedside Index of Severity in Acute Pancreatitis.

^j^PCT: procalcitonin.

^k^PT: prothrombin time.

^l^SVM: support vector machine.

^m^EDT: ensembles of decision trees.

^n^BC: Bayesian classifier.

^o^NLR: neutrophil-lymphocyte ratio.

^p^WBC: white blood cell count.

^q^RF: random forest.

^r^DT: decision tree.

^s^APACHE II: Acute Physiology and Chronic Health Evaluation.

^t^SOFA: Sequential Organ Failure Assessment.

^u^K^+^: potassium.

^v^AMY: blood amylase.

^w^Light GBM: light gradient boosting machine.

^x^AdaBoost: adaptive boosting.

^y^CNB: complement naïve Bayes.

^z^MIMIC-III: Medical Information Mart for Intensive Care III.

^aa^UTHSC: University of Tennessee Health Science Center.

^ab^SpO_2_: peripheral oxygen saturation.

^ac^LDH: lactate dehydrogenase.

^ad^APTT: activated partial thromboplastin time.

^ae^MRR: maximum respiratory rate.

^af^RNN: recurrent neural network.

^ag^NB: naïve Bayes.

^ah^MIMIC IV: Medical Information Mart for Intensive Care IV.

^ai^ICD-9: *International Statistical Classification of Diseases and Related Health Problems, Ninth Revision.*

^aj^ICD-10: *International Statistical Classification of Diseases and Related Health Problems, Tenth Revision.*

^ak^HCO_3_: bicarbonate.

^al^DNN: deep neural networks.

^am^KNN: K-nearest neighbor.

^an^GLMNET: LASSO and elastic net regularized generalized linear models.

^ao^BAYESGLM: Bayesian generalized linear model.

^ap^GAM: generalized additive models.

^aq^GBM: gradient boosting machine.

^ar^NN: neural network.

^as^PNR: pancreatic necrosis rate.

^at^SaO_2_%: arterial blood oxygen saturation.

^au^GBDT: gradient boosting decision trees.

^av^DNN: deep neural network.

#### Feature Selection

While ML excels at handling large datasets, the presence of numerous irrelevant features necessitates effective feature selection. Feature selection is therefore a critical step in developing predictive models and can be categorized into filter, wrapper, and embedded methods [35]. The selection of predictors for each model in the study primarily included demographic data, etiology, past medical history, hemodynamic parameters, laboratory indices, and disease severity scores. The predictors used in the best-performing models across the 17 studies are shown in Table 1. For models with more than 5 predictors, only the top 5 were listed. Among the 17 studies included in the analysis, age emerged as the most prevalent predictive factor, appearing in 11 (64.7%) studies. This was followed by white blood cell count, which was identified as a predictor in 8/17 (47.1%) studies. Respiratory rate was recognized as a predictive factor in 7/17 (41.2%) studies, while arterial oxygen partial pressure or oxygenation index and platelet count were each listed as predictive indicators in 6/17 (35.3%) studies. Additionally, C-reactive protein was identified as an effective predictive marker in 5/17 (29.4%) studies.

#### Quality Assessment

The ROB and the applicability of prediction models were assessed using the PROBAST inventory. Unfortunately, 14 [18-26,28,30,31,33,34] of the included studies exhibited a high ROB (14/17, 82.4%). All 17 studies performed well in terms of study population, showing a low ROB. One study was assessed as having unclear risk in the area of predictors, while the remaining studies were low risk. In the outcome analysis, 6 studies [18,20,21,25,26,34] were considered high risk (6/17, 35.3%), primarily due to the inclusion of predictors directly related to the outcome definition. In total, 5 studies [19,22,24,31,32] were assessed as having unclear risk (5/17, 29.4%) due to insufficient detail on predictor selection or failure to provide time intervals between predictors and outcomes. In data analysis, 11 studies [18-20,22-25,28,30,31,33] were considered high risk (11/17, 64.7%), with the main reasons being insufficient sample size (9 studies), inadequate handling of missing data (8 studies), and the use of univariate analysis for selecting predictors (7 studies). In total, 5 studies [26,27,29,32,34] were assessed as having unclear risk because they did not indicate whether the regression coefficients were consistent with the reported results. Figure S1 in Multimedia Appendix 3 displays the results of the ROB evaluation.

The 17 included studies were well-aligned with the systematic evaluation in terms of the study population, predictors, and findings, and the applicability of the studies was assessed as low risk for applicability concerns.

#### Predicting the Performance of ML Models for ARDS

**Figure 2:**
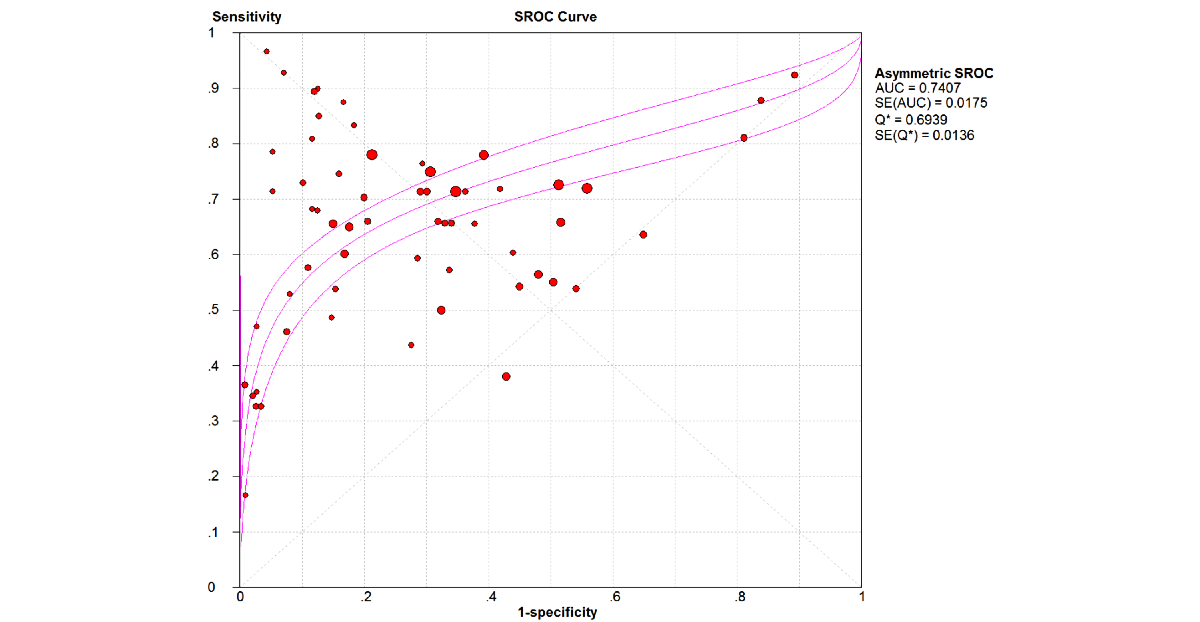
The overall combined area under the receiver operating characteristic curve (AUC) for machine learning models used in acute respiratory distress syndrome prediction. SROC: summary receiver operating characteristic.

**Figure 3:**
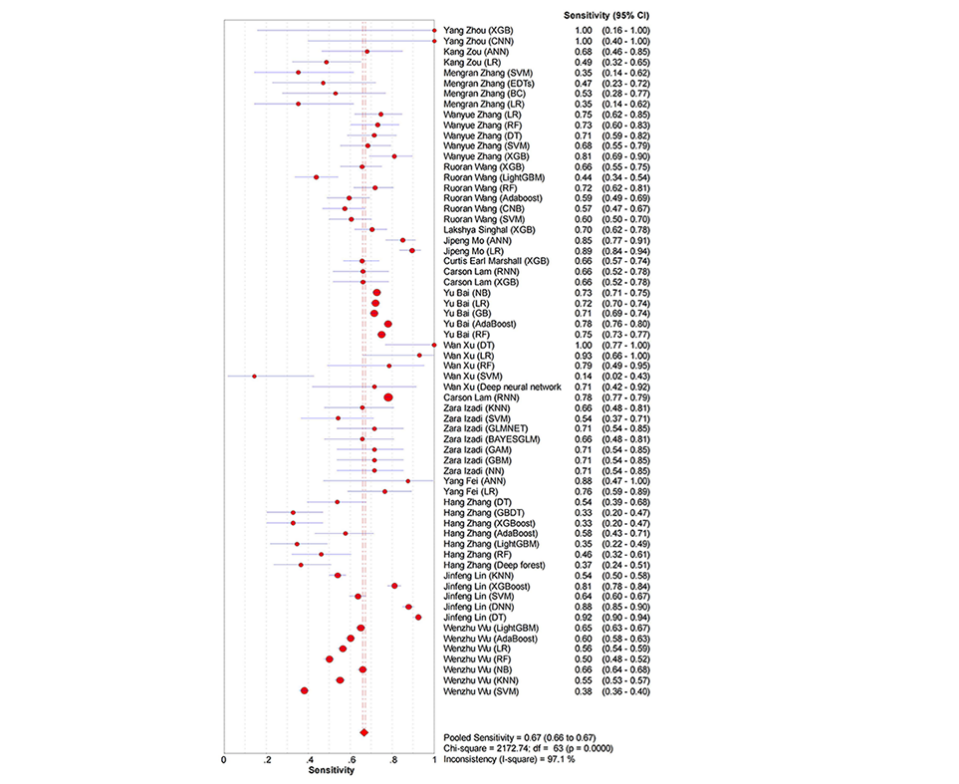
The overall combined sensitivity of machine learning models for predicting acute respiratory distress syndrome [18-34].

**Figure 4:**
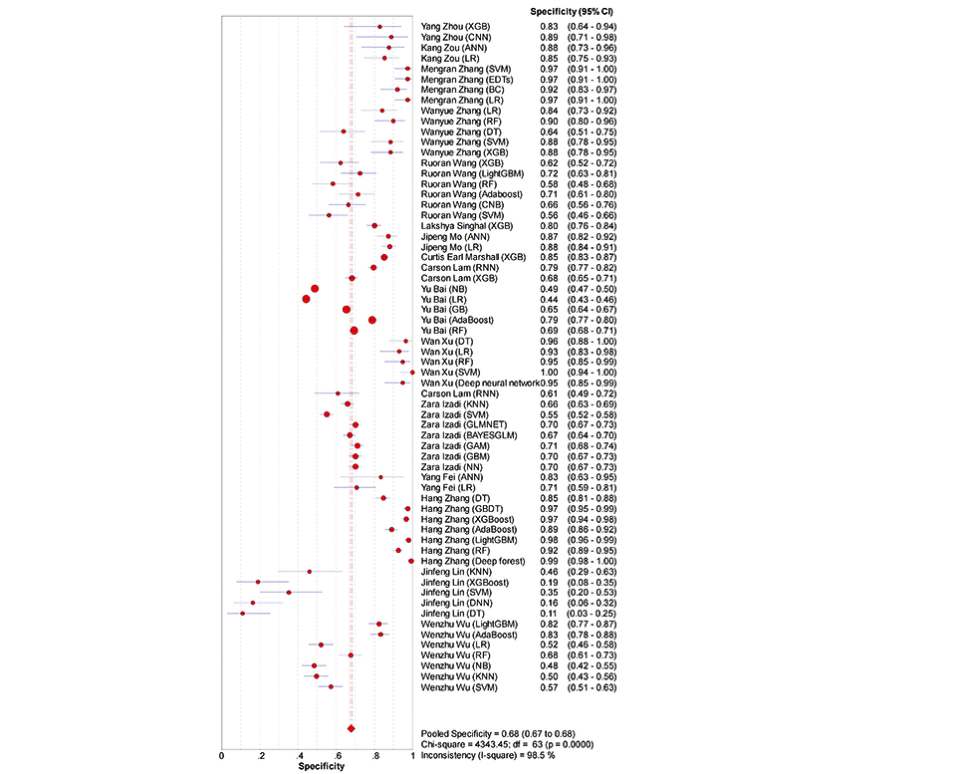
The overall combined specificity of machine learning models for predicting acute respiratory distress syndrome [18-34].

ML models achieved a pooled AUROC of 0.7407 for ARDS (Figure 2). The additional metrics were as follows: sensitivity was 0.67 (95% CI 0.66-0.67; *P*<.001; *I*^2^=97.1%; Figure 3), specificity was 0.68 (95% CI 0.67-0.68; *P*<.001; *I*²=98.5%; Figure 4), the DOR was 6.26 (95% CI 4.93-7.94; *P*<.001; *I*^2^=95.3%; Figure S2 in Multimedia Appendix 3), the PLR was 2.80 (95% CI 2.46-3.19; *P*<.001; *I*^2^=97.3%; Figure S3 in Multimedia Appendix 3), and the NegLR was 0.51 (95% CI 0.46-0.57; *P*<.001; *I*^2^=93.6%; Figure S4 in Multimedia Appendix 3).

#### Sensitivity Analysis

Sensitivity analysis was conducted using Stata software (version 18.0; StataCorp LLC), with the results displayed in Figure S5 (Multimedia Appendix 3). As shown in Figure S5, the results of the sensitivity analysis indicate that among the 64 models, 10 predictive models demonstrated strong sensitivity, while the remaining models did not significantly affect overall sensitivity. Additionally, the distribution of standardized residuals in the figure is relatively concentrated, with no obvious outliers detected. Therefore, the results of the meta-analysis are robust and can be considered reliable.

#### Publication Bias

To assess publication bias, we generated funnel plots using Stata software. The visual inspection of the funnel plots suggested approximate symmetry (*P*=.07). This result indicates that the conclusions of this meta-analysis are robust and not affected by the selective publication of studies (Figure S6 in Multimedia Appendix 3).

#### Subgroup Analysis

This study included a total of 64 predictive models based on over 10 ML algorithms. Table 2 shows the classification of predictive models. To evaluate the performance of the predictive models built using different algorithms, subgroup analyses were performed for those applied more than 3 times. The performance of each algorithm was evaluated using the AUROC, sensitivity, specificity, PLR, NegLR, and DOR, as detailed in Table 3. A total of 14 predictive models were built using the gradient boosting algorithm, yielding a combined AUROC of 0.740. In total, 9 predictive models were based on the ANN algorithm, with an AUROC of 0.8138. In total, 8 models were built using the logistic regression (LR) algorithm, with a combined AUROC of 0.8188. In total, 7 models applied the support vector machine algorithm, with an AUROC of 0.5942. In total, 6 models applied the random forest (RF) algorithm, with an AUROC of 0.7622. Among the predictive models included in the subgroup analysis, the model using the LR algorithm demonstrated the highest AUROC, followed by the ANN algorithm, while the support vector machine algorithm exhibited the lowest performance in terms of AUROC. Additionally, the models exhibited varying performance across different metrics. The decision tree (DT) model achieved the highest sensitivity (0.881, 95% CI 0.855-0.903), the adaptive boosting (Adaboost) model showed the best specificity (0.797, 95% CI 0.785-0.808), the ANN model performed best in terms of DOR (10.671, 95% CI 5.075-22.440).

**Table 2:** Model classification.

Model	Tests, n/N (%)
Gradient boosting decision tree	14/64 (21.9)
Artificial neural network	9/64 (14.1)
Logistic regression	8/64 (12.5）
Support vector machine	7/64 (11)
Random forest	6/64 (9.4)
Bayesian algorithm	5/64 (7.8)
Decision tree	4/64 (6.3)
Adaptive boosting	4/64 (6.3)
K-nearest neighbor	3/64 (4.7)
Ensemble	1/64 (1.7)
Others	3/64 (4.7)

**Table 3:** Model subgroup analysis.

Model	AUROC^a^	Sensitivity, 95% CI	Specificity, 95% CI	PLR^b^, 95% CI	NegLR^c^, 95% CI	DOR^d^, 95% CI)
GB^e^	0.740	0.684 (0.671-0.698)	0.748 (0.739-0.756)	3.333 (2.501-4.441)	0.514 (0.440-0.601)	6.985 (4.809-10.147)
ANN^f^	0.8138	0.793 (0.781-0.806)	0.754 (0.736-0.772)	3.687 (2.126-6.396)	0.345 (0.261-0.455)	10.671 (5.075-22.440)
LR^g^	0.8188	0.657 (0.641-0.672)	0.504 (0.490-0.519）	3.443 (2.145-5.528）	0.429 (0.305-0.603)	9.530 (3.982-22.809)
SVM^h^	0.5942	0.460 (0.441-0.480)	0.605 (0.580-0.631)	1.652 (1.098-2.486)	0.758 (0.581-0.989)	2.833 (1.223-6.565)
RF^i^	0.7622	0.632 (0.616-0.648)	0.715 (0.702-0.727)	3.145 (2.160-4.581)	0.443 (0.291-0.672)	7.625 (3.814-15.242)
Bayesian algorithm	0.6829	0.690 (0.674-0.705)	0.527 (0.513-0.540)	1.616 (1.336-1.954)	0.607 (0.528-0.698)	2.684 (1.935-3.723)
Adaboost^j^	0.8037	0.691 (0.675--0.706)	0.797 (0.785-0.808)	3.486 (2.677-4.538)	0.432 (0.280-0.666)	8.242 (4.870-13.947)
DT^k^	0.7473	0.881 (0.855-0.903)	0.782 (0.745-0.816)	3.129 (1.099-8.906)	0.493 (0.320-0.758)	6.049 (1.938-18.878)

^a^AUROC: area under the receiver operating characteristic curve.

^b^PLR: positive likelihood ratio.

^c^NegLR: negative likelihood ratio.

^d^DOR: diagnostic odds ratio.

^e^GB: gradient boosting.

^f^ANN: artificial neural network.

^g^LR: logistic regression.

^h^SVM: support vector machine.

^i^RF: random forest.

^j^AdaBoost: adaptive boosting.

^k^DT: decision tree.

#### Meta-Regression Analysis

In this study, we conducted a meta-regression analysis to explore potential sources of heterogeneity in predictive model performance. We performed regression analysis by including the publication year (whether the study was published after 2023), sample size (whether the sample size was greater than 1000), the presence of external validation, and the application of LR algorithms or deep learning algorithms. Using Meta Disc software, we sequentially excluded factors such as the application of LR algorithms, deep learning algorithms, and publication year. Through meta-regression analysis, we identified the sources of heterogeneity among studies and assessed their impact on diagnostic outcomes. The results showed that sample size significantly influenced heterogeneity among studies, with diagnostic accuracy tending to decrease as sample size increased (regression coefficient=–1.384, *P*<.001). Additionally, external validation significantly improved diagnostic accuracy, indicating that studies with external validation were more effective in enhancing diagnostic performance (regression coefficient=0.879, *P*=.003). The steps for the meta-regression are in Multimedia Appendix 4.

## Discussion

### Overview

This research aimed to evaluate the effectiveness of different ML algorithms in predicting ARDS. A total of 17 studies were reviewed, which identified 64 different ARDS prediction models. The PROBAST evaluation indicated that most of these studies (14/17, 82.4%) showed a high ROB, mainly due to shortcomings in outcome handling and data analysis. To our knowledge, this is the first systematic review to evaluate ARDS prediction models using multiple effect sizes, including sensitivity, specificity, DOR, PLR, and NegLR. Additionally, we performed a subgroup analysis to compare the predictive accuracy of different ML techniques and explored the sources of heterogeneity among studies.

Clinically, accurate identification and early prediction of ARDS are essential for improving outcomes. Due to limited treatments, research has focused on early detection, with biomarkers and clinical scores like the Lung Injury Prediction Score and Early Acute Lung Injury Score [36]. However, the Lung Injury Prediction Score has a low positive predictive value, and Early Acute Lung Injury Score increases clinical workload without sufficient clinical evidence [37-39].

Biomarkers such as receptors for advanced glycation end products, surfactant protein D, angiopoietin-2, and others are crucial for diagnosis, risk stratification, and identifying ARDS subgroups. Combining angiopoietin-2 with clinical scores improves predictive accuracy. Despite this, biomarkers face clinical challenges like invasiveness and lack of bedside immediacy [40-42]. ML models, especially those using biomarkers, are gaining traction due to their strong predictive performance. However, integrating ML into clinical practice remains difficult due to interpretability issues and lack of physician trust [43,44]. Additionally, although some models have achieved success in research settings, their effectiveness and applicability in real-world clinical environments still require further validation through prospective trials. Future research should focus on overcoming these challenges to facilitate the adoption of ML models in clinical settings, improving patient management.

The subgroup analysis demonstrated that the prediction model using the LR algorithm achieved the highest AUC value, which aligns with the findings reported in a previous study [45]. The reasons for the superior performance of the LR model could be as follows. On the one hand, the dataset used in this study may have been relatively simple or less complex, allowing LR models to perform better compared to more complex models like neural networks. On the other hand, LR models often perform well when the relationships between the predictors and the outcome are linear or nearly linear, which may have been the case in our study.

ANN is a computational model that simulates the interactions of neurons in the brain. It consists of numerous neurons (ie, nodes) interconnected by connections (ie, weights) [46]. ANNs are extensively used in the medical field for biosignal recognition and clinical decision-making. They have been applied in clinical diagnosis for over a decade and have demonstrated the capability to recognize patients more effectively than doctors [46,47]. Our study further demonstrates that ANN algorithms are highly effective in predicting ARDS. RF is a DT-based ML tool adept at handling nonlinearities and missing data, also favored for high-dimensional data analysis, and shows strong predictive performance in this study.

Although AUROC is a commonly used evaluation metric in the field of ML, relying solely on this metric is insufficient to comprehensively assess the overall performance of models. Therefore, this study further examined multiple key metrics across different subgroups. The results showed that the DT model performed best in terms of sensitivity, aiding in the detection rate of positive cases; the Adaboost model demonstrated clear advantages in specificity, exhibiting strong capability in excluding false positives; and the ANN model excelled in DOR, indicating its high diagnostic efficacy in distinguishing between positive and negative cases. These findings highlight the unique strengths of different models in specific clinical applications, suggesting that models should be selected based on specific diagnostic needs in practice to optimize decision support.

### Strengths and Limitations

This study’s advantage lies in its inclusion of nearly all recent ARDS prediction model studies and its evaluation of model performance through various effect sizes, rather than relying solely on the AUROC value. This multidimensional evaluation provides a robust basis for future research. Additionally, unlike previous studies, we conducted a subgroup analysis for prediction algorithms used more than 3 times, aiding in the selection of appropriate and high-performance algorithms. This paper used the PROBAST framework to evaluate the ROB and its applicability in studies focused on predictive modeling. While numerous earlier studies used the Quality Assessment of Diagnostic Accuracy Studies tool, commonly used for estimating bias and applicability in diagnostic accuracy research, PROBAST is better suited for the specific needs of predictive modeling [48]. The PROBAST evaluation revealed that the primary sources of bias are the results and statistical analysis. This indicates that while many studies have reported favorable outcomes, quality assessments have often been overlooked. Future researchers should give greater consideration to predictor selection, the timing of predicted outcomes, and sample size when developing research protocols to mitigate high-bias factors.

Although this study explores the establishment of ARDS prediction models, it has several limitations. First, ARDS is a highly heterogeneous syndrome with distinct subtypes (intrapulmonary and extrapulmonary) that differ in pathophysiology and treatment response. However, due to limited studies, this research did not analyze these subgroups separately, which may introduce selection bias by overlooking potential differences in disease progression and prognosis. Subgroup analysis in future studies could enhance risk stratification and treatment precision.

Second, dataset imbalance and overfitting significantly affect model generalizability. The included studies varied greatly—some were single-center, others multicenter; some included general hospitalized patients, while others focused on specific disease populations. This heterogeneity may lead to models that perform well in certain groups but fail to generalize to others. Furthermore, training models on imbalanced datasets may cause overfitting, where the model becomes too tailored to the training data, reducing its ability to perform well on new, unseen data. Addressing dataset imbalance through representative sampling and using regularization techniques to prevent overfitting could improve robustness and generalizability.

Third, external validation is essential for evaluating model reliability [49]. Many included studies lacked external validation, raising concerns about overfitting. Our meta-regression analysis identified external validation as a key source of heterogeneity and showed that models with external validation had better diagnostic accuracy. Prospective validation is essential to evaluate model performance in real-world clinical workflows. Future research should prioritize both external and prospective validation to enhance the robustness and reliability of predictive models. Prospective validation should be prioritized to ensure real-world applicability.

Finally, while subgroup analyses were conducted for predictive algorithms, data limitations prevented a comprehensive assessment of all models. Future research should explore a wider range of models while ensuring balanced datasets to improve clinical utility.

### Conclusions

This study evaluates prediction models constructed using various ML algorithms, with results showing that ML demonstrates high performance in ARDS prediction. However, many of the existing models still have limitations. During model development, it is essential to focus on model quality, including reducing bias risk, designing appropriate sample sizes, conducting external validation, and ensuring model interpretability. Additionally, challenges such as physician trust and the need for prospective validation must also be addressed. Given that this study is based on a relatively small sample size, larger scale research is needed in the future to comprehensively assess the performance and generalizability of these models. Future research should focus on standardizing model development, optimizing model performance, and exploring how to better integrate predictive models into clinical practice to improve ARDS diagnosis and risk stratification.

## Appendix

Multimedia Appendix 1 PRISMA checklist.

Multimedia Appendix 2 Search strategy.

Multimedia Appendix 3 Supplementary figures.

Multimedia Appendix 4 Meta-regression.

## Abbreviations

AdaBoost: adaptive boosting
ANN: artificial neural network
ARDS: acute respiratory distress syndrome
AUROC: area under the receiver operating characteristic curve
DOR: diagnostic odds ratio
DT: decision tree
ICU: intensive care unit
LR: logistic regression
ML: machine learning
NegLR: negative likelihood ratio
PLR: positive likelihood ratio
PRISMA: Preferred Reporting Items for Systematic Reviews and Meta-Analyses
PROBAST: prediction model risk of bias assessment tool
RF: random forest
ROB: risk of bias
