# Supplementary material for: Predictive Modeling of Acute Respiratory Distress Syndrome Using Machine Learning: Systematic Review and Meta-Analysis
Source: J Med Internet Res. 2025 May 13;27:e66615. doi: 10.2196/66615 (PMC12117268; doi:10.2196/66615)
Supplement: Multimedia Appendix 2 [file jmir_v27i1e66615_app2.doc]

Search strategy

Disease type

Subject Word：Respiratory Distress Syndrome

Random word：

Respiratory Distress Syndromes

Shock Lung

Acute Respiratory Distress Syndrome

ARDS

Human ARDS

Adult Respiratory Distress Syndrom

Intervention measures:

# Subject Word 1：Artificial Intelligence

# Random word 1：

AI

Computer Reasoning

Machine Intelligence

Computational Intelligence

Computer Vision Systems

Computer Vision System

Knowledge Acquisition

Knowledge Representations

Knowledge Representation

Subject Word 2：Deep Learning

Random word 2：Hierarchical Learning

Subject Word 3：Machine Learning

Random word 3：Transfer Learning

# Subject Word 4：Neural Networks, Computer

Random word 4：

Artificial Neural Networks

Artificial Neural Network

Computer Neural Networks

Computer Neural Network

Computational Neural Networks

Computational Neural Network

Neural Network Models

Neural Network Model

Connectionist Models

Connectionist Model

Neural Networks

Neural Network

Perceptrons

Perceptron

Research method：

Prediction

Predictive models

Predictive model

Prediction Models

Prediction Model

1 Pubmed

Disease type:

****((((((Respiratory Distress Syndrome[Title/Abstract]) OR (Respiratory Distress Syndromes[Title/Abstract])) OR (Shock Lung[Title/Abstract])) OR (Acute Respiratory Distress Syndrome[Title/Abstract])) OR (ARDS[Title/Abstract])) OR (Human ARDS[Title/Abstract])) OR (Adult Respiratory Distress Syndrom[Title/Abstract])****

Intervention measures:

Search: ****((((((((((((((((((((((((((((Artificial Intelligence[Title/Abstract]) OR (AI[Title/Abstract])) OR (Computer Reasoning[Title/Abstract])) OR (Machine Intelligence[Title/Abstract])) OR (Computational Intelligence[Title/Abstract])) OR (Computer Vision Systems[Title/Abstract])) OR (Computer Vision System[Title/Abstract])) OR (Knowledge Acquisition[Title/Abstract])) OR (Knowledge Representations[Title/Abstract])) OR (Knowledge Representation[Title/Abstract])) OR (Deep Learning[Title/Abstract])) OR (Hierarchical Learning[Title/Abstract])) OR (Machine Learning[Title/Abstract])) OR (Transfer Learning[Title/Abstract])) OR (Neural Networks, Computer[Title/Abstract])) OR (Artificial Neural Networks[Title/Abstract])) OR (Artificial Neural Network[Title/Abstract])) OR (Computer Neural Networks[Title/Abstract])) OR (Computer Neural Network[Title/Abstract])) OR (Computational Neural Networks[Title/Abstract])) OR (Computational Neural Network[Title/Abstract])) OR (Neural Network Models[Title/Abstract])) OR (Neural Network Model[Title/Abstract])) OR (Connectionist Models[Title/Abstract])) OR (Connectionist Model[Title/Abstract])) OR (Neural Networks[Title/Abstract])) OR (Neural Network[Title/Abstract])) OR (Perceptrons[Title/Abstract])) OR (Perceptron[Title/Abstract])****

Research method：

Search: ****((((Prediction[Title/Abstract]) OR (Predictive models[Title/Abstract])) OR (Predictive model[Title/Abstract])) OR (Prediction Models[Title/Abstract])) OR (Prediction Model[Title/Abstract])****

Outcome:

Search number Query Sort By Filters Search Details Results Time

4 ((((((((Respiratory Distress Syndrome[Title/Abstract]) OR (Respiratory Distress Syndromes[Title/Abstract])) OR (Shock Lung[Title/Abstract])) OR (Acute Respiratory Distress Syndrome[Title/Abstract])) OR (ARDS[Title/Abstract])) OR (Human ARDS[Title/Abstract])) OR (Adult Respiratory Distress Syndrom[Title/Abstract])) AND (((((((((((((((((((((((((((((Artificial Intelligence[Title/Abstract]) OR (AI[Title/Abstract])) OR (Computer Reasoning[Title/Abstract])) OR (Machine Intelligence[Title/Abstract])) OR (Computational Intelligence[Title/Abstract])) OR (Computer Vision Systems[Title/Abstract])) OR (Computer Vision System[Title/Abstract])) OR (Knowledge Acquisition[Title/Abstract])) OR (Knowledge Representations[Title/Abstract])) OR (Knowledge Representation[Title/Abstract])) OR (Deep Learning[Title/Abstract])) OR (Hierarchical Learning[Title/Abstract])) OR (Machine Learning[Title/Abstract])) OR (Transfer Learning[Title/Abstract])) OR (Neural Networks, Computer[Title/Abstract])) OR (Artificial Neural Networks[Title/Abstract])) OR (Artificial Neural Network[Title/Abstract])) OR (Computer Neural Networks[Title/Abstract])) OR (Computer Neural Network[Title/Abstract])) OR (Computational Neural Networks[Title/Abstract])) OR (Computational Neural Network[Title/Abstract])) OR (Neural Network Models[Title/Abstract])) OR (Neural Network Model[Title/Abstract])) OR (Connectionist Models[Title/Abstract])) OR (Connectionist Model[Title/Abstract])) OR (Neural Networks[Title/Abstract])) OR (Neural Network[Title/Abstract])) OR (Perceptrons[Title/Abstract])) OR (Perceptron[Title/Abstract]))) AND (((((Prediction[Title/Abstract]) OR (Predictive models[Title/Abstract])) OR (Predictive model[Title/Abstract])) OR (Prediction Models[Title/Abstract])) OR (Prediction Model[Title/Abstract])) Most Recent ("respiratory distress syndrome"[Title/Abstract] OR "respiratory distress syndromes"[Title/Abstract] OR "shock lung"[Title/Abstract] OR "acute respiratory distress syndrome"[Title/Abstract] OR "ARDS"[Title/Abstract] OR "human ards"[Title/Abstract] OR "adult respiratory distress syndrom"[Title/Abstract]) AND ("artificial intelligence"[Title/Abstract] OR "AI"[Title/Abstract] OR "computer reasoning"[Title/Abstract] OR "machine intelligence"[Title/Abstract] OR "computational intelligence"[Title/Abstract] OR "computer vision systems"[Title/Abstract] OR "computer vision system"[Title/Abstract] OR "knowledge acquisition"[Title/Abstract] OR "knowledge representations"[Title/Abstract] OR "knowledge representation"[Title/Abstract] OR "deep learning"[Title/Abstract] OR "hierarchical learning"[Title/Abstract] OR "machine learning"[Title/Abstract] OR "transfer learning"[Title/Abstract] OR "neural networks computer"[Title/Abstract] OR "artificial neural networks"[Title/Abstract] OR "artificial neural network"[Title/Abstract] OR "computer neural networks"[Title/Abstract] OR "computer neural network"[Title/Abstract] OR "computational neural networks"[Title/Abstract] OR "computational neural network"[Title/Abstract] OR "neural network models"[Title/Abstract] OR "neural network model"[Title/Abstract] OR "connectionist models"[Title/Abstract] OR "connectionist model"[Title/Abstract] OR "neural networks"[Title/Abstract] OR "neural network"[Title/Abstract] OR "Perceptrons"[Title/Abstract] OR "Perceptron"[Title/Abstract]) AND ("Prediction"[Title/Abstract] OR "predictive models"[Title/Abstract] OR "predictive model"[Title/Abstract] OR "prediction models"[Title/Abstract] OR "prediction model"[Title/Abstract]) 117 22:12:37

3 ((((Prediction[Title/Abstract]) OR (Predictive models[Title/Abstract])) OR (Predictive model[Title/Abstract])) OR (Prediction Models[Title/Abstract])) OR (Prediction Model[Title/Abstract]) Most Recent "Prediction"[Title/Abstract] OR "predictive models"[Title/Abstract] OR "predictive model"[Title/Abstract] OR "prediction models"[Title/Abstract] OR "prediction model"[Title/Abstract] 450,073 22:12:23

2 ((((((((((((((((((((((((((((Artificial Intelligence[Title/Abstract]) OR (AI[Title/Abstract])) OR (Computer Reasoning[Title/Abstract])) OR (Machine Intelligence[Title/Abstract])) OR (Computational Intelligence[Title/Abstract])) OR (Computer Vision Systems[Title/Abstract])) OR (Computer Vision System[Title/Abstract])) OR (Knowledge Acquisition[Title/Abstract])) OR (Knowledge Representations[Title/Abstract])) OR (Knowledge Representation[Title/Abstract])) OR (Deep Learning[Title/Abstract])) OR (Hierarchical Learning[Title/Abstract])) OR (Machine Learning[Title/Abstract])) OR (Transfer Learning[Title/Abstract])) OR (Neural Networks, Computer[Title/Abstract])) OR (Artificial Neural Networks[Title/Abstract])) OR (Artificial Neural Network[Title/Abstract])) OR (Computer Neural Networks[Title/Abstract])) OR (Computer Neural Network[Title/Abstract])) OR (Computational Neural Networks[Title/Abstract])) OR (Computational Neural Network[Title/Abstract])) OR (Neural Network Models[Title/Abstract])) OR (Neural Network Model[Title/Abstract])) OR (Connectionist Models[Title/Abstract])) OR (Connectionist Model[Title/Abstract])) OR (Neural Networks[Title/Abstract])) OR (Neural Network[Title/Abstract])) OR (Perceptrons[Title/Abstract])) OR (Perceptron[Title/Abstract]) Most Recent "artificial intelligence"[Title/Abstract] OR "AI"[Title/Abstract] OR "computer reasoning"[Title/Abstract] OR "machine intelligence"[Title/Abstract] OR "computational intelligence"[Title/Abstract] OR "computer vision systems"[Title/Abstract] OR "computer vision system"[Title/Abstract] OR "knowledge acquisition"[Title/Abstract] OR "knowledge representations"[Title/Abstract] OR "knowledge representation"[Title/Abstract] OR "deep learning"[Title/Abstract] OR "hierarchical learning"[Title/Abstract] OR "machine learning"[Title/Abstract] OR "transfer learning"[Title/Abstract] OR "neural networks computer"[Title/Abstract] OR "artificial neural networks"[Title/Abstract] OR "artificial neural network"[Title/Abstract] OR "computer neural networks"[Title/Abstract] OR "computer neural network"[Title/Abstract] OR "computational neural networks"[Title/Abstract] OR "computational neural network"[Title/Abstract] OR "neural network models"[Title/Abstract] OR "neural network model"[Title/Abstract] OR "connectionist models"[Title/Abstract] OR "connectionist model"[Title/Abstract] OR "neural networks"[Title/Abstract] OR "neural network"[Title/Abstract] OR "Perceptrons"[Title/Abstract] OR "Perceptron"[Title/Abstract] 352,477 22:11:54

1 ((((((Respiratory Distress Syndrome[Title/Abstract]) OR (Respiratory Distress Syndromes[Title/Abstract])) OR (Shock Lung[Title/Abstract])) OR (Acute Respiratory Distress Syndrome[Title/Abstract])) OR (ARDS[Title/Abstract])) OR (Human ARDS[Title/Abstract])) OR (Adult Respiratory Distress Syndrom[Title/Abstract]) Most Recent "respiratory distress syndrome"[Title/Abstract] OR "respiratory distress syndromes"[Title/Abstract] OR "shock lung"[Title/Abstract] OR "acute respiratory distress syndrome"[Title/Abstract] OR "ARDS"[Title/Abstract] OR "human ards"[Title/Abstract] OR "adult respiratory distress syndrom"[Title/Abstract] 46,257 22:09:33

2 Embase

Disease type:

'Respiratory Distress Syndrome':ab,ti OR 'Respiratory Distress Syndromes':ab,ti OR 'Shock Lung':ab,ti OR 'Acute Respiratory Distress Syndrome':ab,ti OR 'ARDS':ab,ti OR 'Human ARDS':ab,ti OR 'Adult Respiratory Distress Syndrom':ab,ti

Intervention measures:

'Artificial Intelligence':ab,ti OR 'AI':ab,ti OR 'Computer Reasoning':ab,ti OR 'Machine Intelligence':ab,ti OR 'Computational Intelligence':ab,ti OR 'Computer Vision Systems':ab,ti OR 'Computer Vision System':ab,ti OR 'Knowledge Acquisition':ab,ti OR 'Knowledge Representations':ab,ti OR 'Knowledge Representation':ab,ti OR 'Deep Learning':ab,ti OR 'Hierarchical Learning':ab,ti OR 'Machine Learning':ab,ti OR 'Transfer Learning':ab,ti OR 'Neural Networks, Computer':ab,ti OR 'Artificial Neural Networks':ab,ti OR 'Artificial Neural Network':ab,ti OR 'Computer Neural Networks':ab,ti OR 'Computer Neural Network':ab,ti OR 'Computational Neural Networks':ab,ti OR 'Computational Neural Network':ab,ti OR 'Neural Network Models':ab,ti OR 'Neural Network Model':ab,ti OR 'Connectionist Models':ab,ti OR 'Connectionist Model':ab,ti OR 'Neural Networks':ab,ti OR 'Neural Network':ab,ti OR 'Perceptrons':ab,ti OR 'Perceptron':ab,ti

Research method：

'Prediction':ab,ti OR 'Predictive models':ab,ti OR 'Predictive model':ab,ti OR 'Prediction Models':ab,ti OR 'Prediction Model':ab,ti

Outcome:

Embase

Session Results

.......................................................

No. Query Results Results Date

#4. #1 AND #2 AND #3 124 29 Dec 2024

#3. 'prediction':ab,ti OR 'predictive models':ab,ti 556,745 29 Dec 2024

OR 'predictive model':ab,ti OR 'prediction

models':ab,ti OR 'prediction model':ab,ti

#2. 'artificial intelligence':ab,ti OR 'ai':ab,ti OR 395,797 29 Dec 2024

'computer reasoning':ab,ti OR 'machine

intelligence':ab,ti OR 'computational

intelligence':ab,ti OR 'computer vision

systems':ab,ti OR 'computer vision system':ab,ti

OR 'knowledge acquisition':ab,ti OR 'knowledge

representations':ab,ti OR 'knowledge

representation':ab,ti OR 'deep learning':ab,ti OR

'hierarchical learning':ab,ti OR 'machine

learning':ab,ti OR 'transfer learning':ab,ti OR

'neural networks, computer':ab,ti OR 'artificial

neural networks':ab,ti OR 'artificial neural

network':ab,ti OR 'computer neural

networks':ab,ti OR 'computer neural

network':ab,ti OR 'computational neural

networks':ab,ti OR 'computational neural

network':ab,ti OR 'neural network models':ab,ti

OR 'neural network model':ab,ti OR 'connectionist

models':ab,ti OR 'connectionist model':ab,ti OR

'neural networks':ab,ti OR 'neural network':ab,ti

OR 'perceptrons':ab,ti OR 'perceptron':ab,ti

#1. 'respiratory distress syndrome':ab,ti OR 64,197 29 Dec 2024

'respiratory distress syndromes':ab,ti OR 'shock

lung':ab,ti OR 'acute respiratory distress

syndrome':ab,ti OR 'ards':ab,ti OR 'human

ards':ab,ti OR 'adult respiratory distress

syndrom':ab,ti

.......................................................

3 Cochrane

Disease type:

(Respiratory Distress Syndrome):ti,ab,kw or (Respiratory Distress Syndromes):ti,ab,kw or (Shock Lung):ti,ab,kw or (Acute Respiratory Distress Syndrome):ti,ab,kw or (ARDS):ti,ab,kw or (Human ARDS):ti,ab,kw or (Adult Respiratory Distress Syndrom):ti,ab,kw

Intervention measures:

(Artificial Intelligence):ti,ab,kw or (AI):ti,ab,kw or (Computer Reasoning):ti,ab,kw or (Machine Intelligence):ti,ab,kw or (Computational Intelligence):ti,ab,kw or (Computer Vision Systems):ti,ab,kw or (Computer Vision System):ti,ab,kw or (Knowledge Acquisition):ti,ab,kw or (Knowledge Representations):ti,ab,kw or (Knowledge Representation):ti,ab,kw or (Deep Learning):ti,ab,kw or (Hierarchical Learning):ti,ab,kw or (Machine Learning):ti,ab,kw or (Transfer Learning):ti,ab,kw or (Neural Networks, Computer):ti,ab,kw or (Artificial Neural Networks):ti,ab,kw or (Artificial Neural Network):ti,ab,kw or (Computer Neural Networks):ti,ab,kw or (Computer Neural Network):ti,ab,kw or (Computational Neural Networks):ti,ab,kw or (Computational Neural Network):ti,ab,kw or (Neural Network Models):ti,ab,kw or (Neural Network Model):ti,ab,kw or (Connectionist Models):ti,ab,kw or (Connectionist Model):ti,ab,kw or (Neural Networks):ti,ab,kw or (Neural Network):ti,ab,kw or (Perceptrons):ti,ab,kw or (Perceptron):ti,ab,kw

Research method：

(Prediction):ti,ab,kw or (Predictive models):ti,ab,kw or (Predictive model):ti,ab,kw or (Prediction Models):ti,ab,kw or (Prediction Model):ti,ab,kw

Outcome:

Search Name:

Date Run: 29/12/2024 04:40:16

Comment:

ID Search Hits

#1 (Respiratory Distress Syndrome):ti,ab,kw or (Respiratory Distress Syndromes):ti,ab,kw or (Shock Lung):ti,ab,kw or (Acute Respiratory Distress Syndrome):ti,ab,kw or (ARDS):ti,ab,kw or (Human ARDS):ti,ab,kw or (Adult Respiratory Distress Syndrom):ti,ab,kw 8925

#2 (Artificial Intelligence):ti,ab,kw or (AI):ti,ab,kw or (Computer Reasoning):ti,ab,kw or (Machine Intelligence):ti,ab,kw or (Computational Intelligence):ti,ab,kw or (Computer Vision Systems):ti,ab,kw or (Computer Vision System):ti,ab,kw or (Knowledge Acquisition):ti,ab,kw or (Knowledge Representations):ti,ab,kw or (Knowledge Representation):ti,ab,kw or (Deep Learning):ti,ab,kw or (Hierarchical Learning):ti,ab,kw or (Machine Learning):ti,ab,kw or (Transfer Learning):ti,ab,kw or (Neural Networks, Computer):ti,ab,kw or (Artificial Neural Networks):ti,ab,kw or (Artificial Neural Network):ti,ab,kw or (Computer Neural Networks):ti,ab,kw or (Computer Neural Network):ti,ab,kw or (Computational Neural Networks):ti,ab,kw or (Computational Neural Network):ti,ab,kw or (Neural Network Models):ti,ab,kw or (Neural Network Model):ti,ab,kw or (Connectionist Models):ti,ab,kw or (Connectionist Model):ti,ab,kw or (Neural Networks):ti,ab,kw or (Neural Network):ti,ab,kw or (Perceptrons):ti,ab,kw or (Perceptron):ti,ab,kw 18470

#3 (Prediction):ti,ab,kw or (Predictive models):ti,ab,kw or (Predictive model):ti,ab,kw or (Prediction Models):ti,ab,kw or (Prediction Model):ti,ab,kw 26230

#4 #1 and #2 and #3 12

4 Web of Science

Disease type:

Respiratory Distress Syndrome or Respiratory Distress Syndromes or Shock Lung or Acute Respiratory Distress Syndrome or ARDS or Human ARDS or Adult Respiratory Distress Syndrom

Intervention measures:

Artificial Intelligence or AI or Computer Reasoning or Machine Intelligence or Computational Intelligence or Computer Vision Systems or Computer Vision System or Knowledge Acquisition or Knowledge Representations or Knowledge Representation or Deep Learning or Hierarchical Learning or Machine Learning or Transfer Learning or Neural Networks, Computer or Artificial Neural Networks or Artificial Neural Network or Computer Neural Networks or Computer Neural Network or Computational Neural Networks or Computational Neural Network or Neural Network Models or Neural Network Model or Connectionist Models or Connectionist Model or Neural Networks or Neural Network or Perceptrons or Perceptron

Research method：

Prediction or Predictive models or Predictive model or Prediction Models or Prediction Model

Outcome:

# Web of Science Search Strategy (v0.1)

# Database: Web of Science Core Collection

# Entitlements:

- WOS.IC: 1993 to 2024

- WOS.CCR: 1985 to 2024

- WOS.SCI: 1996 to 2024

- WOS.AHCI: 2002 to 2024

- WOS.ESCI: 2019 to 2024

- WOS.ISTP: 2012 to 2024

- WOS.SSCI: 1982 to 2024

- WOS.ISSHP: 2012 to 2024

# Searches:

1: Respiratory Distress Syndrome or Respiratory Distress Syndromes or Shock Lung or Acute Respiratory Distress Syndrome or ARDS or Human ARDS or Adult Respiratory Distress Syndrom (Topic) AND Artificial Intelligence or AI or Computer Reasoning or Machine Intelligence or Computational Intelligence or Computer Vision Systems or Computer Vision System or Knowledge Acquisition or Knowledge Representations or Knowledge Representation or Deep Learning or Hierarchical Learning or Machine Learning or Transfer Learning or Neural Networks, Computer or Artificial Neural Networks or Artificial Neural Network or Computer Neural Networks or Computer Neural Network or Computational Neural Networks or Computational Neural Network or Neural Network Models or Neural Network Model or Connectionist Models or Connectionist Model or Neural Networks or Neural Network or Perceptrons or Perceptron (Topic) AND Prediction or Predictive models or Predictive model or Prediction Models or Prediction Model (Topic)

Date Run: Sun Dec 29 2024 11:56:34 GMT+0800 (GMT+08:00) Results: 185

5 Scopus

Disease type:

"Respiratory Distress Syndrome" OR "Respiratory Distress Syndromes" OR "Shock Lung" OR "Acute Respiratory Distress Syndrome" OR "ARDS" OR "Human ARDS" OR "Adult Respiratory Distress Syndrom"

Intervention measures:

"Artificial Intelligence" OR "AI" OR "Computer Reasoning" OR "Machine Intelligence" OR "Computational Intelligence" OR "Computer Vision Systems" OR "Computer Vision System" OR "Knowledge Acquisition" OR "Knowledge Representations" OR "Knowledge Representation" OR "Deep Learning" OR "Hierarchical Learning" OR "Machine Learning" OR "Transfer Learning" OR "Neural Networks, Computer" OR "Artificial Neural Networks" OR "Artificial Neural Network" OR "Computer Neural Networks" OR "Computer Neural Network" OR "Computational Neural Networks" OR "Computational Neural Network" OR "Neural Network Models" OR "Neural Network Model" OR "Connectionist Models" OR "Connectionist Model" OR "Neural Networks" OR "Neural Network" OR "Perceptrons" OR "Perceptron"

Research method：

"Prediction" OR "Predictive models" OR "Predictive model" OR "Prediction Models" OR "Prediction Model"

Outcome:

( TITLE-ABS-KEY ( "Respiratory Distress Syndrome" OR "Respiratory Distress Syndromes" OR "Shock Lung" OR "Acute Respiratory Distress Syndrome" OR "ARDS" OR "Human ARDS" OR "Adult Respiratory Distress Syndrom" ) AND TITLE-ABS-KEY ( "Artificial Intelligence" OR "AI" OR "Computer Reasoning" OR "Machine Intelligence" OR "Computational Intelligence" OR "Computer Vision Systems" OR "Computer Vision System" OR "Knowledge Acquisition" OR "Knowledge Representations" OR "Knowledge Representation" OR "Deep Learning" OR "Hierarchical Learning" OR "Machine Learning" OR "Transfer Learning" OR "Neural Networks, Computer" OR "Artificial Neural Networks" OR "Artificial Neural Network" OR "Computer Neural Networks" OR "Computer Neural Network" OR "Computational Neural Networks" OR "Computational Neural Network" OR "Neural Network Models" OR "Neural Network Model" OR "Connectionist Models" OR "Connectionist Model" OR "Neural Networks" OR "Neural Network" OR "Perceptrons" OR "Perceptron" ) AND TITLE-ABS-KEY ( "Prediction" OR "Predictive models" OR "Predictive model" OR "Prediction Models" OR "Prediction Model" ) )

6 ProQuest

Disease type:

AB,TI(''Respiratory Distress Syndrome'' OR ''Respiratory Distress Syndromes'' OR ''Shock Lung'' OR ''Acute Respiratory Distress Syndrome'' OR ''ARDS'' OR ''Human ARDS'' OR ''Adult Respiratory Distress Syndrom'')

Intervention measures:

AB,TI(''Artificial Intelligence'' OR ''AI'' OR ''Computer Reasoning'' OR ''Machine Intelligence'' OR ''Computational Intelligence'' OR ''Computer Vision Systems'' OR ''Computer Vision System'' OR ''Knowledge Acquisition'' OR ''Knowledge Representations'' OR ''Knowledge Representation'' OR ''Deep Learning'' OR ''Hierarchical Learning'' OR ''Machine Learning'' OR ''Transfer Learning'' OR ''Neural Networks, Computer'' OR ''Artificial Neural Networks'' OR ''Artificial Neural Network'' OR ''Computer Neural Networks'' OR ''Computer Neural Network'' OR ''Computational Neural Networks'' OR ''Computational Neural Network'' OR ''Neural Network Models'' OR ''Neural Network Model'' OR ''Connectionist Models'' OR ''Connectionist Model'' OR ''Neural Networks'' OR ''Neural Network'' OR ''Perceptrons'' OR ''Perceptron'')

Research method：

AB,TI(''Prediction'' OR ''Predictive models'' OR ''Predictive model'' OR ''Prediction Models'' OR ''Prediction Model'')

Outcome:

Set#: S1

Searched for: AB,TI(''Respiratory Distress Syndrome'' OR ''Respiratory Distress Syndromes'' OR ''Shock Lung'' OR ''Acute Respiratory Distress Syndrome'' OR ''ARDS'' OR ''Human ARDS'' OR ''Adult Respiratory Distress Syndrom'')

Databases: Business Market Research Collection, Coronavirus Research Database, Ebook Central, History Vault, Periodicals Archive Online, ProQuest Dissertations & Theses Global, ProQuest Historical Newspapers: Chinese Newspapers Collection, Publicly Available Content Database, Research Library

Results: 14685

Set#: S2

Searched for: AB,TI(''Artificial Intelligence'' OR ''AI'' OR ''Computer Reasoning'' OR ''Machine Intelligence'' OR ''Computational Intelligence'' OR ''Computer Vision Systems'' OR ''Computer Vision System'' OR ''Knowledge Acquisition'' OR ''Knowledge Representations'' OR ''Knowledge Representation'' OR ''Deep Learning'' OR ''Hierarchical Learning'' OR ''Machine Learning'' OR ''Transfer Learning'' OR ''Neural Networks, Computer'' OR ''Artificial Neural Networks'' OR ''Artificial Neural Network'' OR ''Computer Neural Networks'' OR ''Computer Neural Network'' OR ''Computational Neural Networks'' OR ''Computational Neural Network'' OR ''Neural Network Models'' OR ''Neural Network Model'' OR ''Connectionist Models'' OR ''Connectionist Model'' OR ''Neural Networks'' OR ''Neural Network'' OR ''Perceptrons'' OR ''Perceptron'')

Databases: Business Market Research Collection, Coronavirus Research Database, Ebook Central, History Vault, Periodicals Archive Online, ProQuest Dissertations & Theses Global, ProQuest Historical Newspapers: Chinese Newspapers Collection, Publicly Available Content Database, Research Library

Results: 884728

Set#: S3

Searched for: AB,TI(''Prediction'' OR ''Predictive models'' OR ''Predictive model'' OR ''Prediction Models'' OR ''Prediction Model'')

Databases: Business Market Research Collection, Coronavirus Research Database, Ebook Central, History Vault, Periodicals Archive Online, ProQuest Dissertations & Theses Global, ProQuest Historical Newspapers: Chinese Newspapers Collection, Publicly Available Content Database, Research Library

Results: 769375

Set#: S4

Searched for: [S1] AND [S2] AND [S3]

Databases: Business Market Research Collection, Coronavirus Research Database, Ebook Central, History Vault, Periodicals Archive Online, ProQuest Dissertations & Theses Global, ProQuest Historical Newspapers: Chinese Newspapers Collection, Publicly Available Content Database, Research Library

These databases are searched for part of your query.

Results: 68

----------------------------------------------------------
