# Supplementary material for: Predictive Modeling of Acute Respiratory Distress Syndrome Using Machine Learning: Systematic Review and Meta-Analysis
Source: J Med Internet Res. 2025 May 13;27:e66615. doi: 10.2196/66615 (PMC12117268; doi:10.2196/66615)
Supplement: Multimedia Appendix 3 [file jmir_v27i1e66615_app3.docx]

Multimedia Appendix 3

##### Figure S1：Risk of bias assessment result.


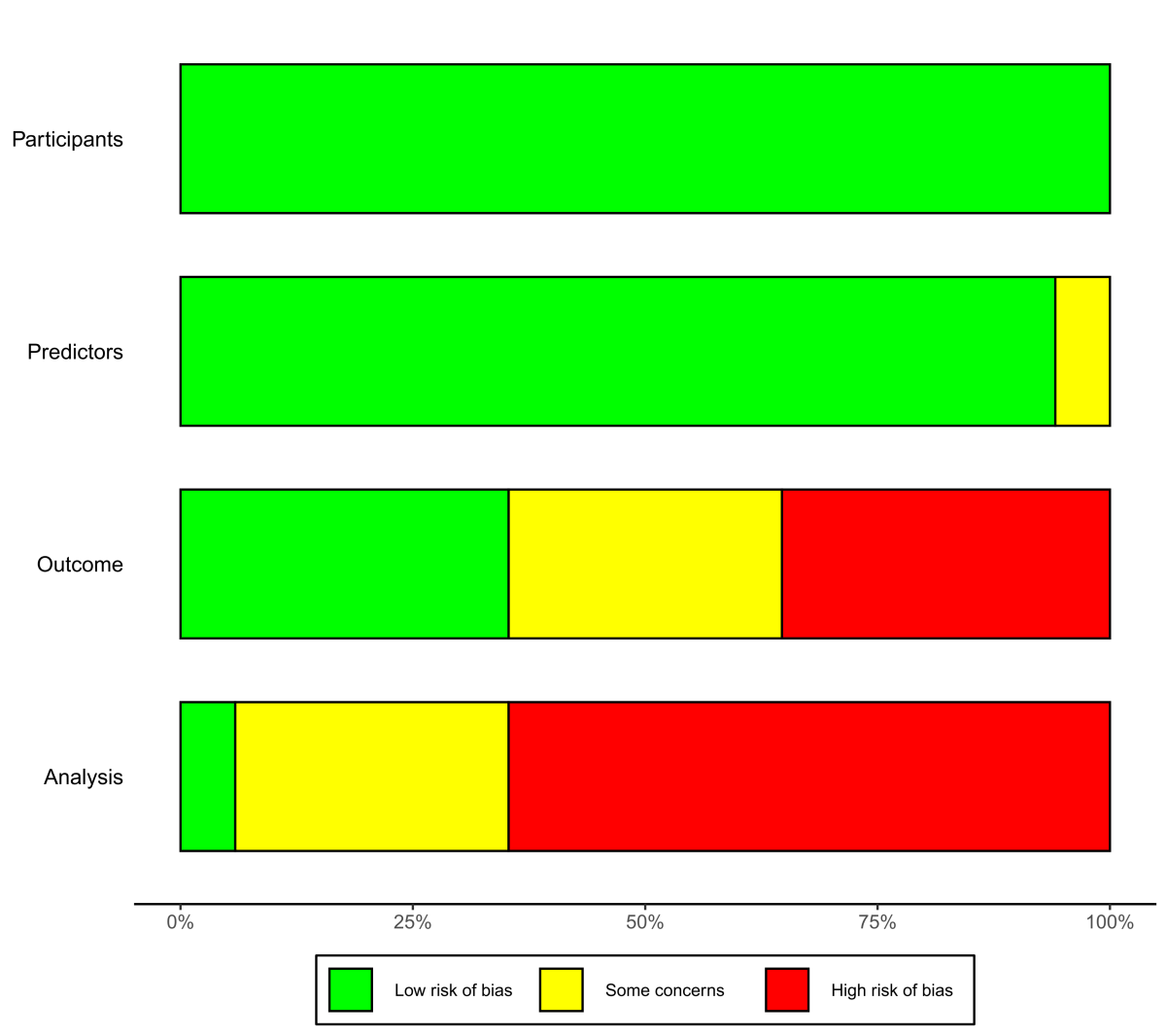


##### Figure S2：The overall aggregated diagnostic odds ratio for machine learning models predicting acute respiratory distress syndrome.

#####
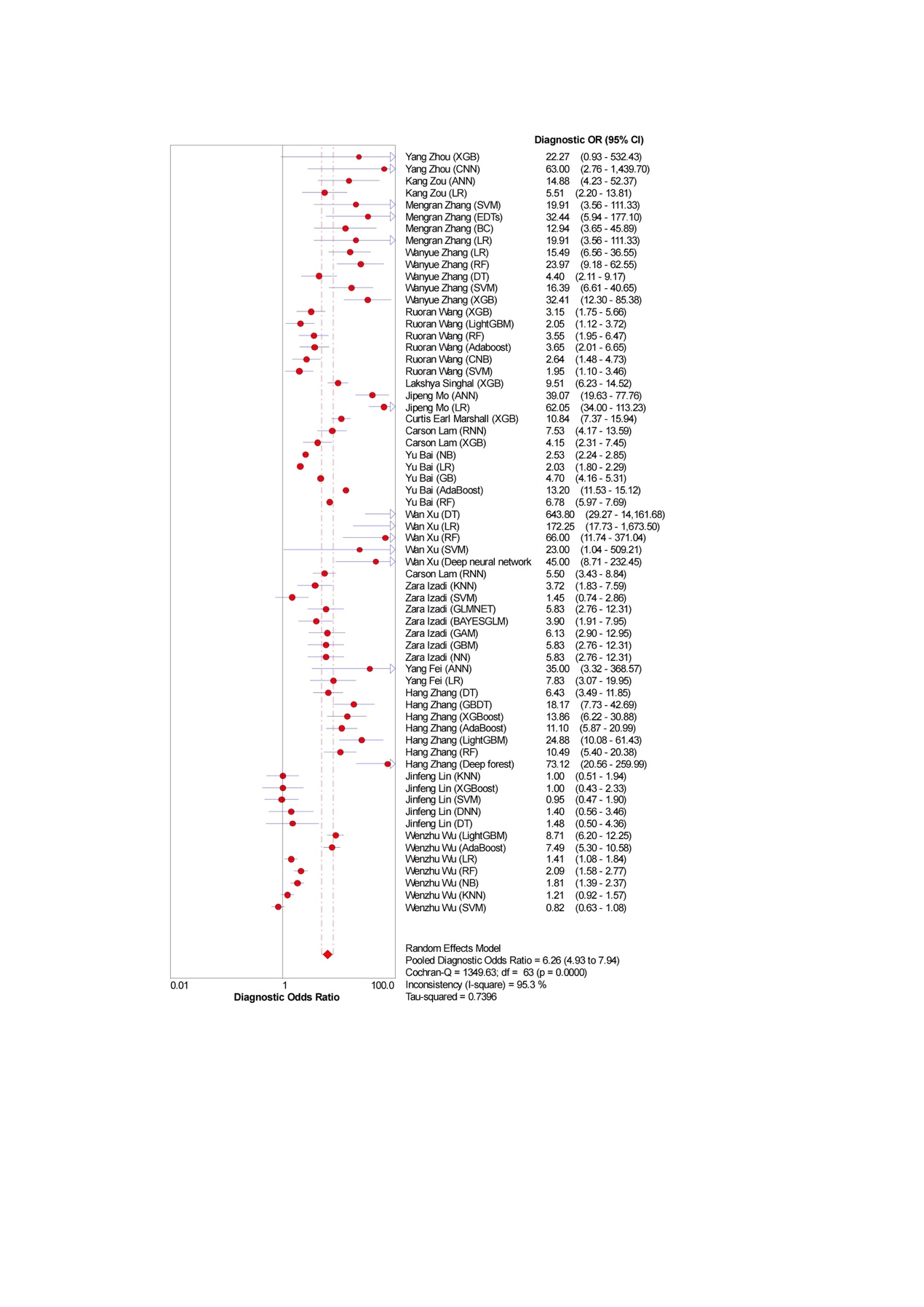


##### Figure S3 The overall positive likelihood ratio of machine learning for acute respiratory distress syndrome prediction.


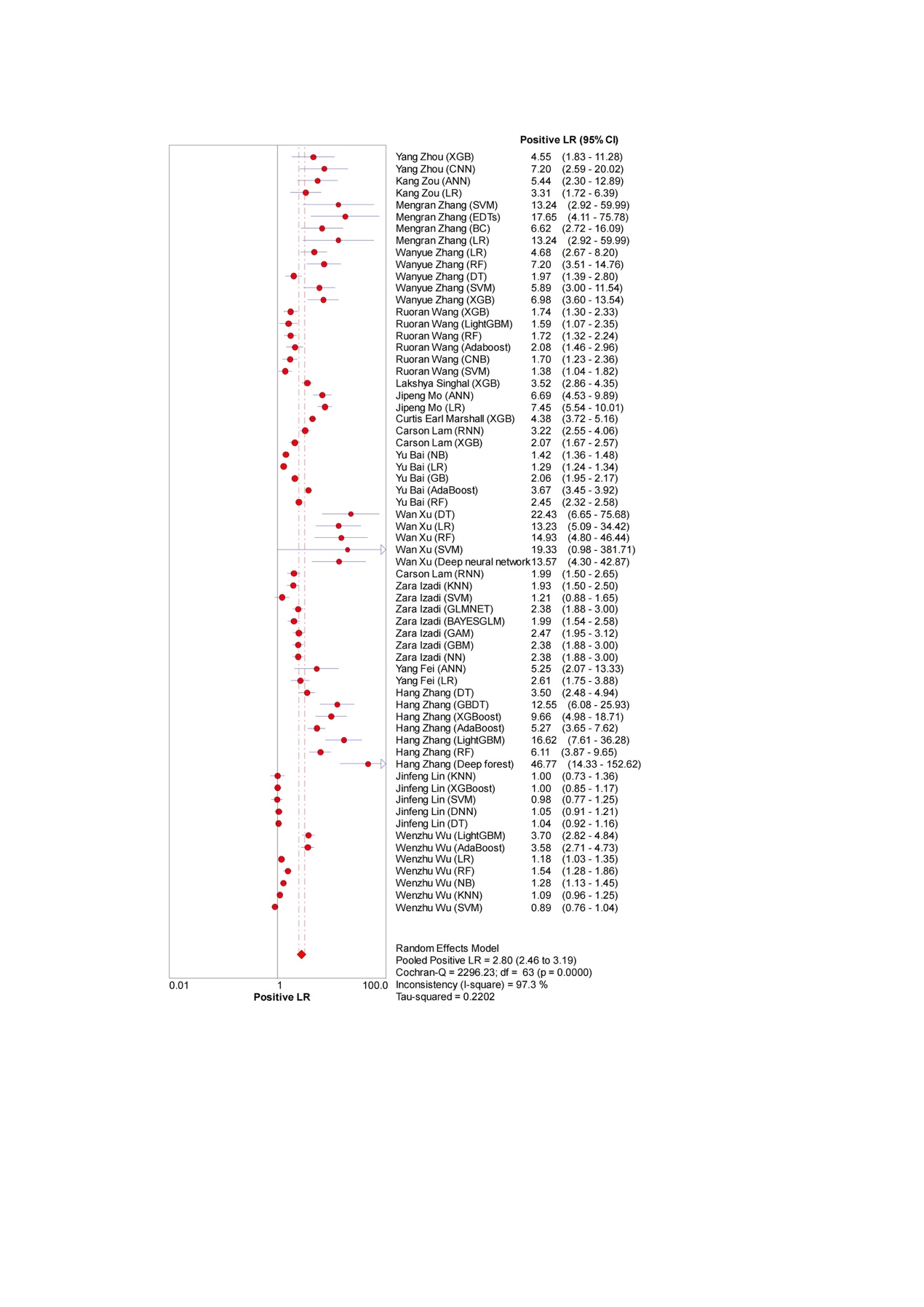


##### Figure S4 The overall negative likelihood ratio of machine learning for acute respiratory distress syndrome prediction.


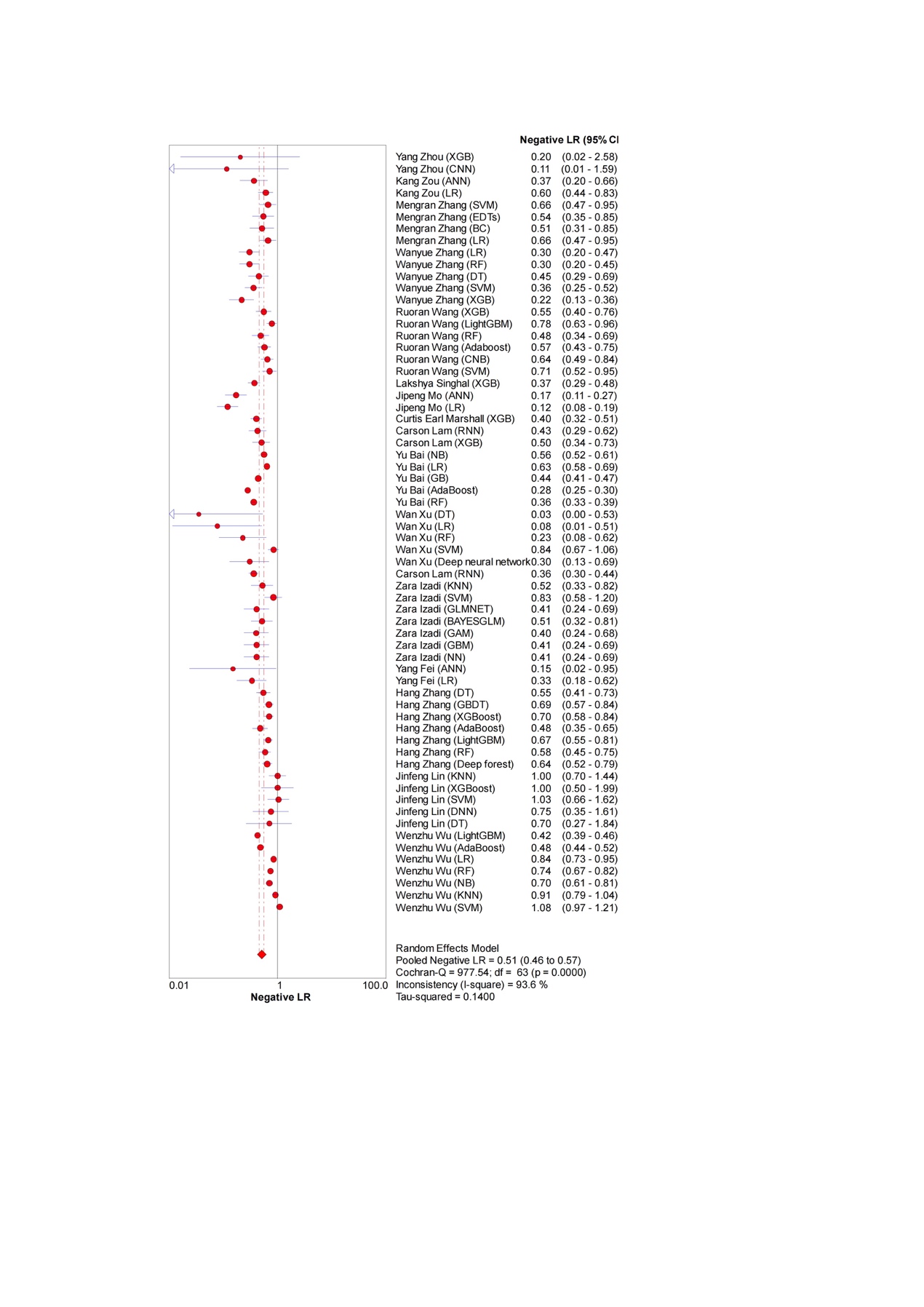


##### Figure S5 Sensitivity analysis

#####
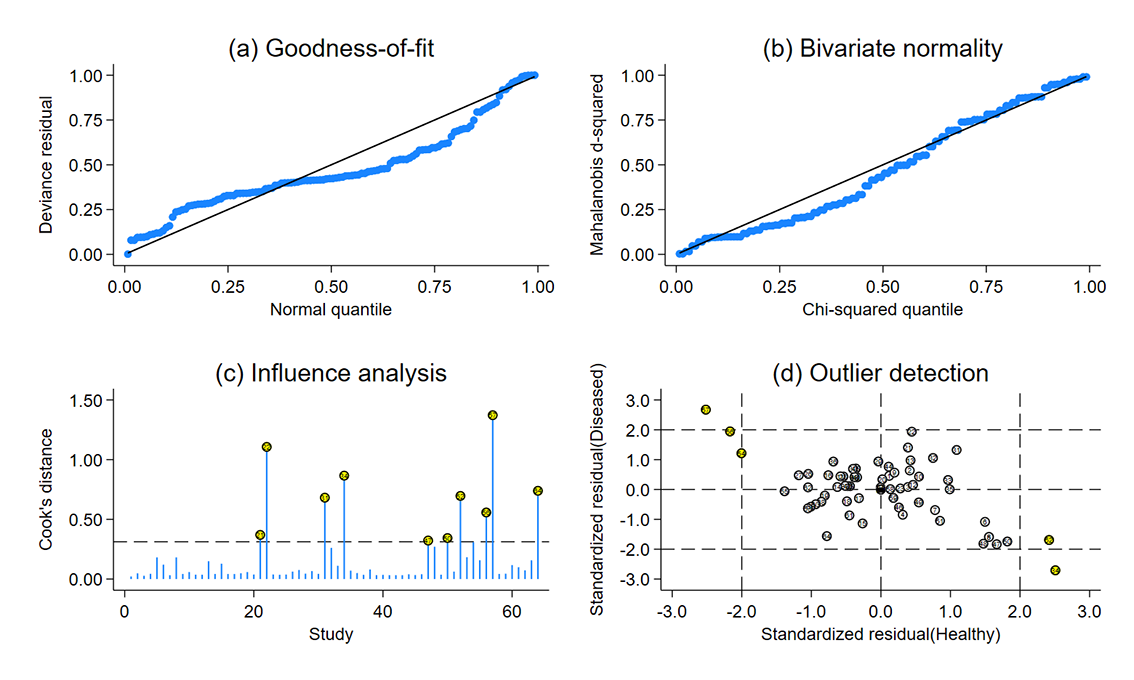


##### Figure S6 Publication bias funnel plot


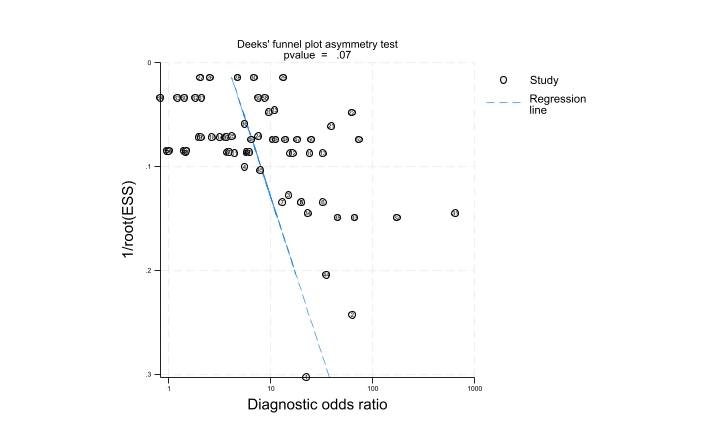


ESS: Effective sample size
