# Supplementary material for: Predictive Modeling of Acute Respiratory Distress Syndrome Using Machine Learning: Systematic Review and Meta-Analysis
Source: J Med Internet Res. 2025 May 13;27:e66615. doi: 10.2196/66615 (PMC12117268; doi:10.2196/66615)
Supplement: Multimedia Appendix 4 [file jmir_v27i1e66615_app4.docx]

#### Multimedia Appendix 4: Results of Meta-regression

Meta-regression is employed to explore potential sources of heterogeneity by examining the relationship between study-level characteristics and effect sizes. This approach extends traditional meta-analysis by incorporating covariates that may influence the observed outcomes. In this study, we applied a weighted regression model, where the dependent variable is the log-transformed diagnostic odds ratio (DOR), and independent variables represent key study characteristics. The regression coefficients and their standard errors were estimated using the ordinary or weighted least squares method, with weights based on either sample size or the inverse variance of the log-transformed DOR. This analysis allows for the identification of factors contributing to variations in diagnostic performance across studies, thereby providing a more comprehensive understanding of the influence of different study conditions on the results.

| Variety | Coeff. | Std. Err. | p – value | RDOR | [95%CI] |
| --- | --- | --- | --- | --- | --- |
| Cte. | 2.242 | 0.3156 | 0.0000 | ---- | ---- |
| S | -0.388 | 0.0650 | 0.0000 | ---- | ---- |
| Year | -0.428 | 0.2609 | 0.1060 | 0.65 | (0.39;1.10) |
| Size | -1.508 | 0.3460 | 0.0001 | 0.22 | (0.11;0.44) |
| Verification | 0.854 | 0.2814 | 0.0036 | 2.35 | (1.34;4.13) |
| LR | 0.278 | 0.3473 | 0.4265 | 1.32 | (0.66;2.65) |
| DL | 0.649 | 0.3476 | 0.0671 | 1.91 | (0.95;3.84) |

| Variety | Coeff. | Std. Err. | p – value | RDOR | [95%CI] |
| --- | --- | --- | --- | --- | --- |
| Cte. | 2.343 | 0.2885 | 0.0000 | ---- | ---- |
| S | -0.384 | 0.0647 | 0.0000 | ---- | ---- |
| Year | -0.461 | 0.2572 | 0.0783 | 0.63 | (0.38;1.06) |
| Size | -1.568 | 0.3369 | 0.0000 | 0.21 | (0.11;0.41) |
| Verification | 0.845 | 0.2806 | 0.0039 | 2.33 | (1.33;4.08) |
| DL | 0.602 | 0.3420 | 0.0836 | 1.83 | (0.92;3.62) |

| Variety | Coeff. | Std. Err. | p – value | RDOR | [95%CI] |
| --- | --- | --- | --- | --- | --- |
| Cte. | 2.473 | 0.2842 | 0.0000 | ---- | ---- |
| S | -0.365 | 0.0650 | 0.0000 | ---- | ---- |
| Year | -0.504 | 0.2605 | 0.0581 | 0.60 | (0.36;1.02) |
| Size | -1.688 | 0.3359 | 0.0000 | 0.18 | (0.09;0.36) |
| Verification | 0.942 | 0.2809 | 0.0014 | 2.56 | (1.46;4.50) |

| Variety | Coeff. | Std. Err. | p – value | RDOR | [95%CI] |
| --- | --- | --- | --- | --- | --- |
| Cte. | 2.075 | 0.1967 | 0.0000 | ---- | ---- |
| S | -0.343 | 0.0652 | 0.0000 | ---- | ---- |
| Size | -1.384 | 0.3005 | 0.0000 | 0.25 | (0.14;0.46) |
| Verification | 0.879 | 0.2847 | 0.0031 | 2.41 | (1.36;4.26) |
